# Benchmarking comes of age

**DOI:** 10.1186/s13059-019-1846-5

**Published:** 2019-10-09

**Authors:** Mark D. Robinson, Olga Vitek

**Affiliations:** 1 Institute of Molecular Life Sciences and SIB Swiss Institute of Bioinformatics, University of Zurich, 8057 Zurich, Switzerland; 2 Khoury College of Computer Sciences, Northeastern University, Boston, MA USA

The emergence of microarrays in the late 1990s, the introduction of massively parallel nucleotide sequencing in the mid 2000s, and the continuous improvement of mass spectrometric and imaging technologies transformed the way we practice biology. The new technologies produce datasets of ever increasing complexity and size, and in turn stimulate massive advances in all areas of data science. A vast repertoire of bespoke statistical and computational methods and software tools now process, interpret, and condense these datasets, to help generate and test new hypotheses and extract actionable insight.

This diversity of the methods and tools presents its own challenge. Alternative views of a problem give rise to multiple distinct tools for a same general purpose. Even small differences in data preprocessing or analysis in these tools can impact the downstream biological conclusions, and even produce contradictory results. The fast pace of biotechnology and data science makes it increasingly difficult to choose a right technology, and a right data analysis method or tool, for the scientific question at hand. These challenges feed broader misunderstandings among the consumers of the scientific research in the age of concerns for irreproducibility, science skepticism, and fake news.

This special issue takes the view that rigorous and exhaustive benchmarking is an effective way to address these challenges. Since not all methods and tools stand the test of time [1–3], they must be vetted beyond the original publication [4]. In post-genomic biology, such vetting includes documenting the limitations of data produced by various biotechnologies, the fidelity of the computational transformations and preprocessing of the data, the validity of statistical modeling assumptions, and the availability (when legally allowed) of the open source code and data. Benchmarking efforts [5, 6] aim to perform such vetting in a transparent manner, and highlight the gaps in methodology and in implementations.

Proper and conclusive benchmarking of methods and tools has its own methodological challenges, and *Genome Biology* has long been an active proponent of benchmarking research [7, 8]. As the first articles from this special issue now appear in print, several known and new themes addressing the methodological challenges of benchmarking have emerged.

In some cases, benchmarking is made challenging by the sheer number of approaches available. For example, Kosugi et al. [9] investigated 69 structural variation detection algorithms; Abdelaal et al. [10] evaluated 22 single cell classification methods across 27 datasets; and Zielezinksi et al. [11] tested 74 alignment methods. The high variation in installability of bioinformatics tools alone observed by Mangul et al. [12] made such comparisons a daunting task.

Beyond considering the set of relevant methods for a particular task, an important aspect of benchmarking is the choice of reference datasets [13]. Most often, benchmarks combine synthetic and experimental datasets. On the one hand, simulations provide an opportunity to scrutinize methods across a wide set of conditions, such as the level of informativeness of a covariate used to weight the false discovery budget [14] or to the true underlying evolutionary selection scenarios [15], while relying on ground truth. Such simulations must demonstratively retain the key properties of the experimental data [13]. On the other hand, the experimental datasets must contain some notion of a ground truth, established at “arm’s length” from the methods evaluated. For example, Wick et al. relied on an accurate reference genome sequence [16] base calling of Nanopore data; Abdelaal et al. used pre-sorted cell populations or manual annotation to compare cell-level classification methods [10]; and Mendoza et al. evaluated the reconstruction of metabolic networks using manually curated models [17].

Given reference datasets with ground truth and a set of methods to test, the choice of metrics for method evaluation is non-trivial, and often context-specific. For example, the performance of base calling can be reported at either read level or consensus level, their relative importance depending on the application [16]. Another example is F1 score, a classical metric for evaluating cell type assignment [10]. However, the metric does not characterize the performance by cell type, and benchmarkers balance detail and resolution at their discretion. Mendoza et al. created a suite of 22 ad hoc features to evaluate the properties of tools for reconstructing metabolic networks [17]. Finally, although run times are somewhat of peripheral significance, they become important when many competing methods perform well.

Although the benchmarks in this issue are rigorous and comprehensive, they only reflect the state of the art at the current point in time. But the scientific advances do not stop. More methodological research and infrastructure must be developed in the future for *dynamic* addition of new methods, reference datasets, and metrics to the benchmarks. This will enable an early integration of an emerging benchmark into the method developers community, will avoid the “method explosion” as in the analysis of single cell data [18] or the “evaluation explosion” where every new method is evaluated differently [11], and will preserve the relevance of the benchmarks in time.

The objectives of dynamic evaluation are facilitated by the availability of open data and code. For example, Duò and Soneson made available data and software for adding new clustering algorithms directly to existing figures [19]. Furthermore, the broader benchmarking community could adopt practices of challenge-based benchmarks, such as containerization and code validation [20]. These practices may be particularly valuable even for benchmarks that do not require controlled “model-to-data” access, but still benefit from cloud computing and continuous “frictionless” integration [20]. Extensibility with new datasets and metrics will also be welcome. Such openness will increase the credibility of the benchmarking process and will maximize its impact.
